# Association of age at menopause and suicide risk in postmenopausal women: a nationwide cohort study

**DOI:** 10.3389/fpsyt.2024.1442991

**Published:** 2024-12-17

**Authors:** Daa Un Moon, Hyewon Kim, Jin-Hyung Jung, Kyungdo Han, Hong Jin Jeon

**Affiliations:** ^1^ Department of Psychiatry and Neurosciences, Charité Campus Mitte (CCM), Charité Universitätsmedizin Berlin, Berlin, Germany; ^2^ Department of Psychiatry and Psychotherapy, Psychiatric University Hospital Charité at St. Hedwig Hospital, Berlin, Germany; ^3^ Department of Psychiatry, Depression Center, Samsung Medical Center, Sungkyunkwan University School of Medicine, Seoul, Republic of Korea; ^4^ Department of Psychiatry, Hallym University Sacred Heart Hospital, Anyang, Republic of Korea; ^5^ Samsung Biomedical Research Institute, Sungkyunkwan University School of Medicine, Suwon, Republic of Korea; ^6^ Department of Statistics and Actuarial Science, Soongsil University, Seoul, Republic of Korea; ^7^ Department of Health Sciences and Technology, Department of Medical Device Management and Research, and Department of Clinical Research Design and Evaluation, Samsung Advanced Institute for Health Sciences and Technology (SAIHST), Sungkyunkwan University, Seoul, Republic of Korea

**Keywords:** age at menopause, suicide, primary ovarian insufficiency, early menopause, postmenopausal women, menopause

## Abstract

**Introduction:**

Early age at menopause has been linked to various adverse health outcomes, but its association with suicide risk remains underexplored. This study aims to assess the relationship between age at menopause and suicide risk among postmenopausal women.

**Methods:**

This retrospective cohort study analyzed data from the Korean National Health Insurance System (NHIS), covering 1,315,795 postmenopausal women aged 30 years and above, from 2009 to 2021. Menopausal age was classified as primary ovarian insufficiency (under 40 years), early menopause (40-44 years), average menopause (45-49 and 50-54 years), and late menopause (55 years and older). Suicide incidence was identified using ICD-10 codes for primary cause of death. Multivariable Cox proportional hazards models were utilized to estimate hazard ratios (HRs) and 95% confidence intervals (CIs).

**Results:**

Across the 12-year follow-up, there were 2,986 suicides. Women with primary ovarian insufficiency exhibited the highest suicide risk (HR, 1.43; 95% CI, 1.14–1.78, *p* < 0.001), followed by those with early menopause (HR, 1.31; 95% CI, 1.15–1.50, *p* < 0.001), and those with menopause between 45 and 49 (HR, 1.13; 95% CI, 1.04–1.23, *p* < 0.001) compared to the reference group undergoing menopause at age of 50-54.

**Discussion:**

Early onset of menopause, particularly primary ovarian insufficiency, is associated with a significantly elevated risk of suicide. These findings underscore the need for targeted interventions and support for women experiencing early menopause. This study highlights the importance of monitoring mental health in postmenopausal women and suggests further research to explore the underlying mechanisms linking early menopause to increased suicide risk.

## 1 Introduction

Menopause, defined as the cessation of menstruation for 12 consecutive months, marks a pivotal transition in a woman’s life (1). Occurring at a median age of 51.4 years (2), this period is characterized by hormonal changes, including declining estrogen levels and rising follicle-stimulating hormone (FSH) concentrations, signaling the end of reproductive capacity (3). Beyond physical symptoms such as hot flashes and vaginal dryness, menopause impacts mental health, with hormonal fluctuations linked to an array of psychiatric comorbidities, including depression, cognitive decline, and insomnia (4–6). The perimenopausal period marked by estrogen fluctuations is associated with an increased risk of depression (7). The decline in estrogen disrupts stress response and emotional regulation, potentially exacerbating depressive symptoms (8). Moreover, estrogen acts as a neuroprotective factor, influencing brain function and protecting against cognitive decline. Although the precise mechanisms are still under investigation, the decline in estrogen during menopause may disrupt these protective systems, potentially leading to an increased vulnerability to mental health problems (9).

The timing of menopause has been shown to affect mental health: Primary ovarian insufficiency (POI), defined as the depletion or dysfunction of ovarian follicles with cessation of menses before the age of 40, affects about 1% of women globally and 2.4% in South Korean (10) (hereafter referred to as Korea). Characterized by premature ovarian function decline, earlier cessation of menstruation is associated with several health risks (11). The etiology of POI is complex and varied, including genetic factors, autoimmune disorders, environmental exposures, and impact of medical treatments such as chemotherapy and radiation. However, the main mechanism remains unknown in most cases (12). Women with POI face increased risks of cardiovascular disease (13), osteoporosis (14), mental health conditions including depression and anxiety (15, 16), and cognitive decline (17). Psychosocial effects of POI are significant, often resulting in grief, anxiety, and depression. They are amplified by societal stigma and uncertainties surrounding the disorder (18). Notably, a previous study conducted in Korea has linked POI to an increased tendency towards suicidal thoughts (19). These findings indicate a connection between early menopause and elevated mental health risks, including suicide.

Despite the recognized impact of menopause on mental health, the potential connection between timing of menopause and suicide risk remains underexplored. Suicide remains a significant global health concern, accounting for over 700,000 deaths annually, highlighting the urgency of addressing all potential risk factors (20). Its gravity is particularly pronounced in Korea, which has been reported to have the highest suicide rate among OECD countries from 2003 to 2020, with 24.1 suicides per 100,000 persons (21). While various biological, psychological, clinical, and socio-environmental determinants have been investigated, it is crucial to continue exploring under-recognized or emerging risk factors that may contribute to mental health vulnerability in women (22).

Our study aimed to explore the association between menopausal timing and suicide risk among postmenopausal women, leveraging the comprehensive dataset from the Korean National Health Insurance System (NHIS). In addition to established risk factors such as depression and chronic physical conditions, we included detailed reproductive data, such as parity and breastfeeding duration, due to their potential impact on hormonal stability and mental health. Previous studies suggest that reproductive factors, including parity (23) and breastfeeding (24), may influence estrogen and progesterone levels, thereby affecting neuroendocrine pathways involved in mood regulation and mental health (25, 26). By investigating a comprehensive set of reproductive and health-related covariates, we aim to provide a nuanced understanding of how menopausal timing may influence suicide risk, contributing to a broader understanding of menopause’s impact on women’s mental health. We hypothesize that earlier onset of menopause is associated with an increased risk of suicide, thus contributing to a broader understanding of menopause’s impact on women’s health.

## 2 Methods and analysis

### 2.1 Study design and data source

This study was a retrospective cohort analysis utilizing a database provided by the NHIS. The NHIS is a single-payer, mandatory, comprehensive health insurance program covering 97% of Korea’s population, while the remaining 3% is covered by the Medical Aid Program for low-income individuals (27). The NHIS database includes a broad range of population-based health data, such as demographic information, health examination records, disease diagnoses, and treatment data coded according to the International Classification of Diseases, Tenth Revision, Clinical Modification (ICD-10-CM) (28). Since 2002, the NHIS has continuously compiled this information, enabling reliable population-level research for nearly all residents in Korea (29). The NHIS provides biennial health screenings for all insured individuals, including laboratory tests and self-reported questionnaires on health behaviors (30). This extensive dataset allows for representative analyses of health outcomes across the Korean population.

### 2.2 Study population

**Figure 1:**
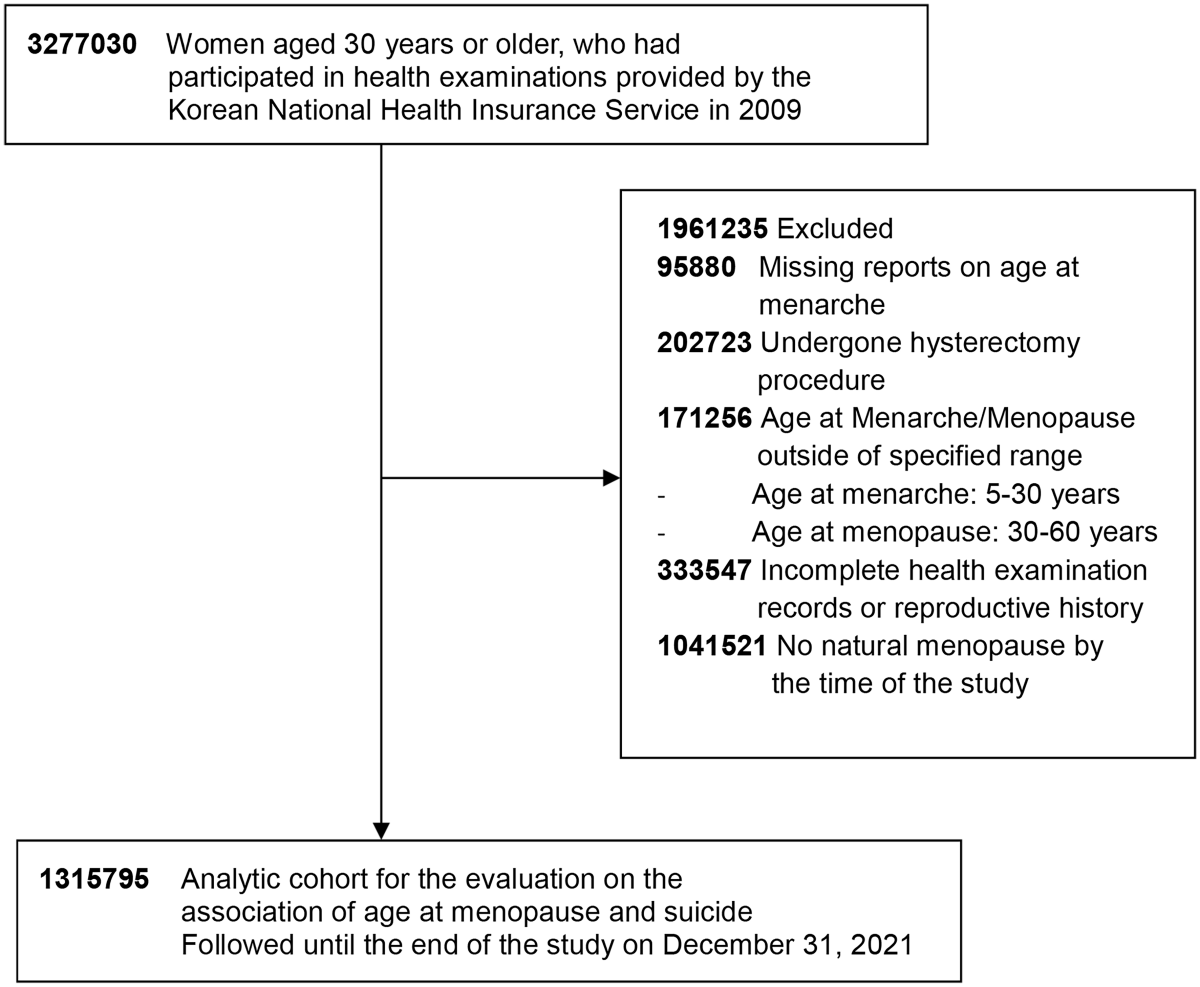
Flowchart showing the selection of study population.

The initial cohort included 3,277,030 women aged 30 years and older who had participated in health examinations provided in 2009. We excluded those without a reported age at menarche (*n* = 95,880) and those who had undergone hysterectomy procedures (*n* = 202,723). Women with reported ages at menarche and menopause outside the specified range of 5 to under 30 years for menarche and 30 to under 60 years for menopause were also excluded (*n* = 171,256). Additional exclusions were made for individuals with incomplete health examination records or reproductive history (*n* = 333,547) and for those who had not experienced natural menopause by the time of the study (*n* = 1,041,521). After these criteria were applied, our final cohort for this study consisted of 1,315,795 postmenopausal women (**Figure 1**).

### 2.3 Study endpoint and follow-up

The primary outcome, death by suicide, was ascertained through linkage with data from Statistics Korea, the National Statistical Office of Korea. Causes of death were determined using ICD-10 codes (X60-X84) for suicide recorded as the primary cause of death in the database. Selected individuals were followed up until the end of the study on December 31, 2021. The mean follow-up duration was 12 years, starting from the baseline health check-up in 2009 to the end of the study period or the occurrence of suicide.

### 2.4 Menopausal status

In this study, menopause was defined as permanent cessation of menstrual periods, which is determined retrospectively following a span of 12 consecutive months of amenorrhea. Menopause typically occurs between ages of 45 and 55 years. We categorized postmenopausal women into four groups based on their age at the onset of menopause: under 40 years (POI), 40-44 years (early menopause), 45-49 years and 50-54 years (both falling in the normal range), and those aged ≥ 55 years.

### 2.5 Covariates

Our study was adjusted for multiple covariates, including detailed reproductive factors using standardized self-reporting questionnaires. Age at menarche was categorized into four groups: 5−12, 13−14, 15−16, and ≥ 17 years. Parity (0, 1, ≥ 2 children), duration of oral contraceptive use (none, < 1 year, ≥ 1 year), breastfeeding duration (none, < 6 months, 6 months up to < 1 year, ≥ 1 year), and use of HRT (none, < 2 years, 2 to 5 years, ≥ 5 years) were also recorded. The duration of fertility was calculated as the interval between menarche and menopause. It was categorized as < 30, 30-34, 35-39, and ≥ 40 years.

Demographic information, such as age and sex, was taken into consideration during the analysis. Economic status was determined based on health insurance premiums, which are indicative of income level in Korea. Participants were categorized as having a low income if they received medical aid or fell within the bottom 25% income bracket versus those outside of this category. Lifestyle factors were obtained from self-reported questionnaires completed by participants during health screenings. Smoking behavior was divided into current smokers and non-smokers. Alcohol consumption was assessed by categorizing individuals as drinkers or non-drinkers based on their self-reported intake frequency. Physical activity was classified into two categories: 1) those engaging in regular moderate-intensity exercise for at least 30 minutes on 5 or more days per week or vigorous activity for at least 20 minutes on 3 or more days per week, and 2) those not engaging such exercise. Obesity was defined by a body mass index (BMI) ≥ 25 kg/m^2^ (31). Other clinical measures included waist circumference, blood pressure, and serum levels of glucose and cholesterol.

Baseline comorbidities in our study were determined through a combination of ICD-10, pharmacy records, and physical examination findings, supplemented by medical service claims data. Hypertension was determined by either a claim for antihypertensive medication under codes I10−I13 and I15 or recorded blood pressure readings ≥ 140/90 mmHg. Type 2 diabetes was confirmed by at least one annual claim for antidiabetic medication under codes E11−E14 or fasting glucose level ≥ 126 mg/dL. Dyslipidemia was defined as having at least one yearly claim for lipid-lowering medication under code E78 or total cholesterol level ≥ 240 mg/dL. Chronic kidney disease was classified based on an estimated glomerular filtration rate below 60 mL/min/1.73 m^2^, calculated according to the Modification of Diet in Renal Disease study equation (32) under the code N18 or N19. Depression was identified using codes F32 and F33, anxiety under codes F40, and F41 and schizophrenia under F20. The selected covariates encompass reproductive, lifestyle, and mental health-related factors relevant to menopausal timing and suicide risk.

### 2.6 Statistical analysis

Baseline characteristics are described using means ± standard deviations for continuous variables and frequencies with percentages for categorical variables, stratified by age at menopause. Comparison of continuous variables across groups was performed using analysis of variance, while the χ^2^ test was employed for categorical variables. Incidence rates of suicide were calculated as the number of events per 1,000 person-years of follow-up. Kaplan-Meier curves were generated to depict cumulative event rates of suicide by age at menopause.

The association between age at menopause and the risk of suicide were analyzed using multivariable Cox proportional hazards regression, yielding hazard ratios (HRs), and 95% confidence intervals (CIs). Prior to performing the Cox proportional hazards regression, we confirmed that the proportional hazards assumption was met. Model 1 was unadjusted. Model 2 was adjusted for age. Model 3 was further adjusted for socioeconomic and lifestyle factors such as income, smoking status, alcohol consumption, and physical activity. Model 4 was further adjusted for clinical factors, including obesity, type 2 diabetes, hypertension, dyslipidemia, chronic kidney disease, and depression. Model 5 extended adjustments to include reproductive factors, including age at menarche, parity, breastfeeding history, use of oral contraceptives, and hormone replacement therapy (HRT). We included an additional model, Model 6, that extends Model 5 by adjusting for schizophrenia and anxiety to account for a broader range of mental health factors.

All statistical tests were two-sided, and a *P*-value of less than 0.05 was considered to indicate statistical significance. All statistical analyses were performed using SAS software version 9.4 (SAS Institute Inc.).

This study was conducted following the principles of the Declaration of Helsinki. It was approved by the Institutional Review Board (IRB) of Soongsil University (No. SSU-202007-HR-236-01). Given the use of de-identified data, informed consent was waived by the IRB. The study design and analysis adhered to the Strengthening the Reporting of Observational Studies in Epidemiology (STROBE) reporting guideline (33).

## 3 Results

### 3.1 Baseline characteristics of the study population

The study population’s baseline characteristics are summarized in **Table 1**. The study cohort comprised 1,315,795 postmenopausal women with a mean age of 61.7 years. Mean menarche occurred at 16.46 years and mean menopause at 49.96 years. Of the study cohort, 1.8% had POI with an average onset at 36.75 years old. Lifestyle factors varied by age at menopause. Younger menopausal age was associated with a higher rate of smoking (4.2%) but a lower rate of regular exercise (15.4%). Comorbidities such as chronic kidney disease (15.2%) and depression (7.95%) were more prevalent in those with POI. Differences across menopause age groups were statistically significant (*P* < 0.001) for all comparisons.

**Table 1:** Baseline characteristics of the study population by age at menopause.

	TOTAL (*N* = 1315795)	Age at Menopause (years)					*P* value
		<40 (*n* = 23452)	40-44 (*n* = 77099)	45-49 (*n* = 360032)	50-54 (*n* = 718110)	55- (*n* = 137102)	
Age (years)	61.69 ± 8.45	63.73 ± 10.35	63.06 ± 10.53	60.9 ± 9.25	61.48 ± 8.02	63.77 ± 5.98	<0.001
Low income^a^	281723 (21.41)	4794 (20.44)	15948 (20.69)	76718 (21.31)	154932 (21.57)	29331 (21.39)	<0.001
Current smoker^b^	34523 (2.62)	985 (4.2)	2821 (3.66)	10982 (3.05)	17000 (2.37)	2735 (1.99)	<0.001
Alcohol drinker^c^	159798 (12.14)	2720 (11.6)	9324 (12.09)	46491 (12.91)	86370 (12.03)	14893 (10.86)	<0.001
Regular exerciser^d^	241463 (18.35)	3613 (15.41)	12166 (15.78)	63784 (17.72)	133341 (18.57)	28559 (20.83)	<0.001
Obesity^e^	490293 (37.26)	9172 (39.11)	28867 (37.44)	127653 (35.46)	264023 (36.77)	60578 (44.18)	<0.001
Type 2 Diabetes	171317 (13.02)	3723 (15.87)	11108 (14.41)	44199 (12.28)	90614 (12.62)	21673 (15.81)	<0.001
Hypertension	561558 (42.68)	10967 (46.76)	34502 (44.75)	145039 (40.29)	302900 (42.18)	68150 (49.71)	<0.001
Dyslipidemia	427437 (32.49)	7559 (32.23)	23638 (30.66)	111374 (30.93)	234974 (32.72)	49892 (36.39)	<0.001
Chronic kidney disease	157875 (12)	3567 (15.21)	11406 (14.79)	43897 (12.19)	81761 (11.39)	17244 (12.58)	<0.001
Depression	91888 (6.98)	1864 (7.95)	6032 (7.82)	25488 (7.08)	48843 (6.80)	9661 (7.05)	<0.001
Anxiety	183797(13.97)	3826(16.31)	11903(15.44)	50168(13.93)	98727(13.75)	19173(13.98)	<.0001
Schizophrenia	1889(0.14)	50(0.21)	142(0.18)	565(0.16)	977(0.14)	155(0.11)	<.0001
Height (cm)	153.46 ± 5.73	152.22 ± 5.99	152.5 ± 6.1	153.47 ± 5.83	153.6 ± 5.68	153.43 ± 5.38	<0.001
Weight (kg)	56.99 ± 8.30	56.25 ± 8.94	56.16 ± 8.82	56.64 ± 8.37	57.05 ± 8.19	58.22 ± 8.13	<0.001
BMI (kg/m^2^)	24.18 ± 3.16	24.24 ± 3.38	24.12 ± 3.35	24.03 ± 3.19	24.16 ± 3.11	24.71 ± 3.10	<0.001
Waist circumference (cm)	80.07 ± 8.29	80.92 ± 8.78	80.4 ± 8.75	79.67 ± 8.41	79.94 ± 8.18	81.51 ± 8.04	<0.001
Fasting glucose (mg/dL)	99.76 ± 24.40	100.74 ± 26.56	100.3 ± 25.48	99.31 ± 23.97	99.57 ± 24.2	101.53 ± 25.46	<0.001
Systolic BP (mmHg)	125.7 ± 16.17	126.66 ± 16.81	126.17 ± 16.73	125.02 ± 16.29	125.58 ± 16.04	127.66 ± 15.98	<0.001
Diastolic BP (mmHg)	76.94 ± 10.16	77.44 ± 10.4	77.15 ± 10.37	76.69 ± 10.18	76.92 ± 10.12	77.56 ± 10.1	<0.001
Total cholesterol (mg/dL)	207.74 ± 38.8	205.93 ± 39.53	205.56 ± 39.2	206.97 ± 38.72	208.26 ± 38.69	208.56 ± 39.14	<0.001
Age at Menopause (years)	49.96 ± 3.98	36.75 ± 2.59	41.80 ± 1.50	47.43 ± 1.41	51.35 ± 1.38	56.14 ± 1.42	<0.001
Age at Menarche (years)	16.46 ± 1.84	16.87 ± 2.11	16.64 ± 2.03	16.42 ± 1.85	16.39 ± 1.79	16.77 ± 1.88	<0.001
< 13	12789 (0.97)	328 (1.40)	1013 (1.31)	4105 (1.14)	6444 (0.90)	899 (0.66)	
13-14	159700 (12.14)	2737 (11.67)	10253 (13.3)	48401 (13.44)	85130 (11.85)	13179 (9.61)	
15-16	509871 (38.75)	6800 (29)	25063 (32.51)	134788 (37.44)	296123 (41.24)	47097 (34.35)	
≥ 17	633435 (48.14)	13587 (57.94)	40770 (52.88)	172738 (47.98)	330413 (46.01)	75927 (55.38)	
Duration of fertility (years)	33.50 ± 4.38	19.88 ± 3.30	25.17 ± 2.65	31.02 ± 2.35	34.96 ± 2.23	39.37 ± 2.27	<0.001
< 30	185463 (14.10)	23438 (99.94)	73397 (95.2)	84839 (23.56)	3730 (0.52)	59 (0.04)	
30-34	551506 (41.91)	14 (0.06)	3667 (4.76)	253817 (70.5)	292409 (40.72)	1599 (1.17)	
35-39	497956 (37.84)	.	35 (0.05)	21180 (5.88)	406387 (56.59)	70354 (51.32)	
≥ 40	80870 (6.15)	.	.	196 (0.05)	15584 (2.17)	65090 (47.48)	
Parity	1293811 (98.33)	22824 (97.32)	75404 (97.8)	353146 (98.09)	707071 (98.46)	135366 (98.73)	<0.001
Nulliparity	21984 (1.67)	628 (2.68)	1695 (2.2)	6886 (1.91)	11039 (1.54)	1736 (1.27)	
1	77793 (5.91)	1645 (7.01)	5011 (6.5)	24861 (6.91)	40376 (5.62)	5900 (4.3)	
≥ 2	1216018 (92.42)	21179 (90.31)	70393 (91.3)	328285 (91.18)	666695 (92.84)	129466 (94.43)	
Duration of breastfeeding (months)	1231650 (93.61)	21592 (92.07)	71153 (92.29)	333196 (92.55)	675324 (94.04)	130385 (95.1)	<0.001
Never	84145 (6.39)	1860 (7.93)	5946 (7.71)	26836 (7.45)	42786 (5.96)	6717 (4.9)	
< 6	82115 (6.24)	1194 (5.09)	4740 (6.15)	25224 (7.01)	44799 (6.24)	6158 (4.49)	
6 – 12	222667 (16.92)	2830 (12.07)	10548 (13.68)	60524 (16.81)	128923 (17.95)	19842 (14.47)	
≥ 12	926868 (70.44)	17568 (74.91)	55865 (72.46)	247448 (68.73)	501602 (69.85)	104385 (76.14)	
Duration of OC use (years)	203285 (15.45)	3232 (13.78)	11466 (14.87)	56327 (15.64)	106726 (14.86)	25534 (18.62)	<0.001
Never	1112510 (84.55)	20220 (86.22)	65633 (85.13)	303705 (84.36)	611384 (85.14)	111568 (81.38)	
< 1	122096 (9.28)	1992 (8.49)	6920 (8.98)	33891 (9.41)	64660 (9)	14633 (10.67)	
≥ 1	81189 (6.17)	1240 (5.29)	4546 (5.9)	22436 (6.23)	42066 (5.86)	10901 (7.95)	
Duration of HRT (years)	210086 (15.97)	4172 (17.79)	13616 (17.66)	65418 (18.17)	104476 (14.55)	22404 (16.34)	<0.001
Never	1105709 (84.03)	19280 (82.21)	63483 (82.34)	294614 (81.83)	613634 (85.45)	114698 (83.66)	
< 2	122029 (9.27)	1865 (7.95)	7106 (9.22)	37317 (10.36)	63254 (8.81)	12487 (9.11)	
2-5	49963 (3.8)	932 (3.97)	3184 (4.13)	15701 (4.36)	24537 (3.42)	5609 (4.09)	
≥5	38094 (2.9)	1375 (5.86)	3326 (4.31)	12400 (3.44)	16685 (2.32)	4308 (3.14)	

Data are presented as mean ± standard deviation or number (%).

BMI, body mass index; BP, blood pressure; HRT, hormone replacement therapy; OC, oral contraceptives.

a Low income: Individuals in the bottom 25% income bracket based on health insurance premiums in South Korea.

b Current smoker: Includes individuals who currently smoke, as opposed to non-smokers (never-smokers and ex-smokers).

c Alcohol Drinker: Individuals reporting any frequency of alcohol consumption.

d Regular Exerciser: Individuals engaging in moderate-intensity exercise for at least 30 minutes on 5+ days per week, or vigorous activity for at least 20 minutes on 3+ days per week.

e Obesity: Body mass index (BMI) of 25 kg/m² or higher.

SI conversion factors: to convert glucose to millimoles per liter, multiply by 0.0555; high-density lipoprotein cholesterol to millimoles per liter, multiply by 0.0259; low-density lipoprotein cholesterol to millimoles per liter, multiply by 0.0259; total cholesterol to millimoles per liter, multiply by 0.0259.

### 3.2 Mortality distribution across menopausal age groups

**Table 2** outlines data on total mortality and follow-up duration categorized by menopausal age. Among the cohort, a total of 134,021 (10.19%) deaths were recorded, with the highest occurrence observed in women with POI (16.38%). Suicide accounted for 2,986 (0.23%) of all deaths. Again, the highest rate of suicide was observed in the under 40 group (0.35%). The average follow-up period for the entire cohort was approximately 11.96 ± 1.83 years. A slightly longer duration was seen in the group who experienced menopause after age of 55 years (12.02 ± 1.72 years). Median durations remained consistent at around 12.41 years across all age categories examined. Statistically significant variations in mortality rates were evident across different ages at menopause (*P* < 0.001).

**Table 2:** Mortality and follow-up duration by age at menopause.

	TOTAL	Age at Menopause (years)					
		<40	40-44	45-49	50-54	55-	*P* value
Subjects (n)	1315795	23452	77099	360032	718110	137102	
Total mortality (%)	134021 (10.19)	3842 (16.38)	11724 (15.21)	37395 (10.39)	68448 (9.53)	12612 (9.2)	<0.001
Suicide (%)	2986 (0.23)	83 (0.35)	242 (0.31)	866 (0.24)	1506 (0.21)	289 (0.21)	<0.001
Follow Up Duration (person-year)							
Mean ± SD	11.96 ± 1.83	11.73 ± 2.24	11.75 ± 2.19	11.96 ± 1.84	11.98 ± 1.78	12.02 ± 1.72	<0.001
Median (Q1-Q3)	12.41 (12.1-12.69)	12.44 (12.08-12.75)	12.42 (12.08-12.73)	12.41 (12.11-12.69)	12.4 (12.1-12.68)	12.41 (12.11-12.68)	<0.001

Table displays mortality and suicide rates (%) along with follow-up duration (person-years) among 1,315,795 subjects categorized by age at menopause (< 40 to 55+ years). Data show total counts, percentages, and follow-up metrics, with statistical significance noted by p values.

### 3.3 Incidence and risk of suicide according to age at menopause

**Figure 2:**
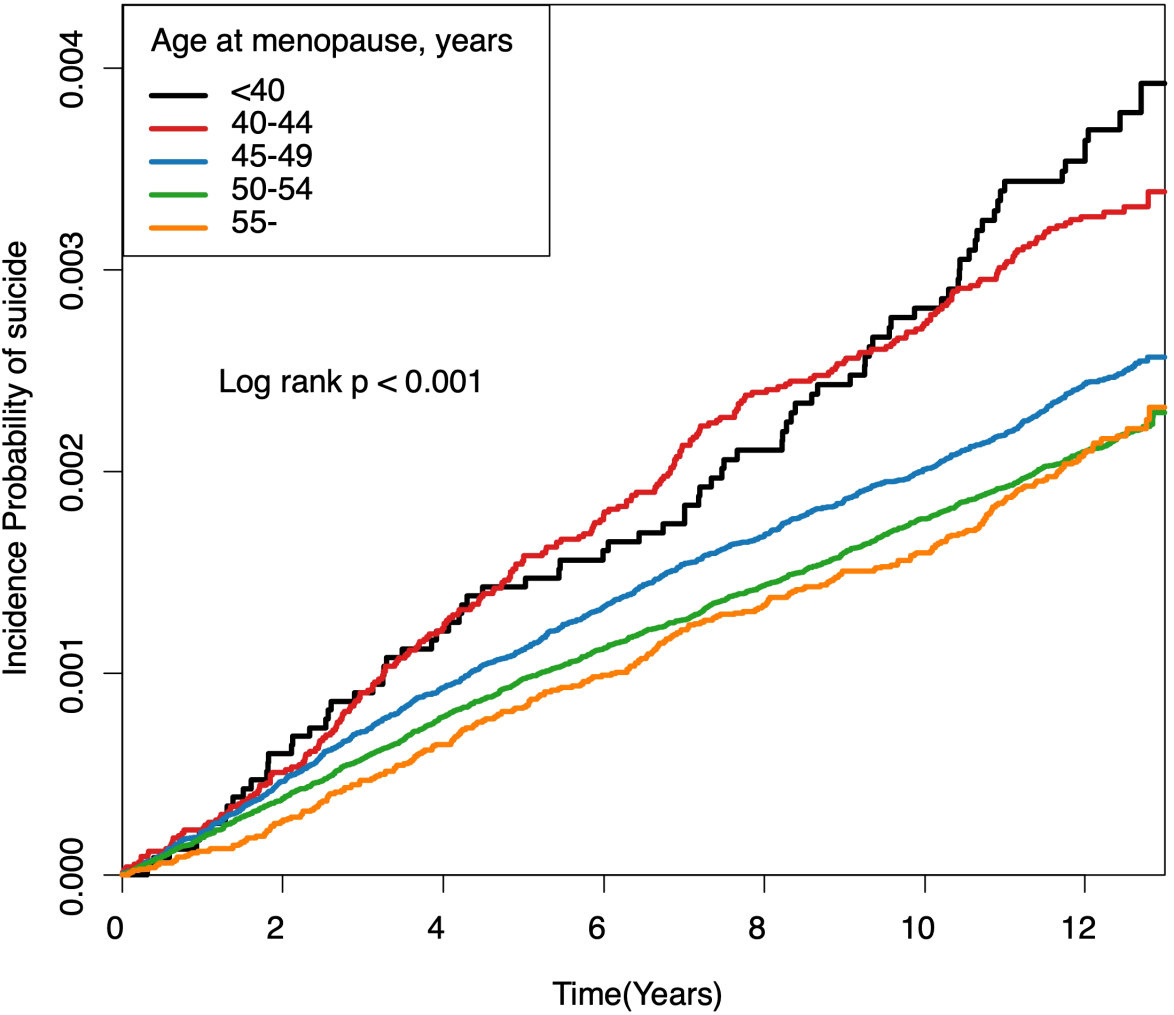
Cumulative event rates of suicide associated with age at menopause. Kaplan-Meier estimates for cumulative incidence of suicide over a 12-year period, stratified by age at menopause. Each line represents an age category: under 40, 40-44, 45-49, 50-54, and 55 years and older. Log-rank tests indicate significant differences among groups (all log-rank *P*-Values < 0.001).

**Figure 2** illustrates cumulative event rates of suicide, stratified by menopausal age group, with rates increasing inversely with age at menopause. This trend was statistically significant across all groups (*p* < 0.001). Incidence rates and HRs for suicide, as detailed in **Table 3**, varied with age at menopause. Women with POI exhibited the highest incidence rate of 0.30 per 1000 person-years and faced a 42.8% increased risk of suicide (HR, 1.428; 95% CI, 1.144-1.782) compared to the reference group (50-54 years) even after adjusting for covariates including demographic, lifestyle, clinical and reproductive factors in Model 5. Relative to the reference group, those in the 40-44 age range had a 31.2% increased risk of suicide (HR, 1.312; 95% CI, 1.145-1.503), while those in the age range of 45-49 had a 13.4% increased risk (HR, 1.134; 95% CI, 1.043-1.233). For women aged 55 and older, the risk was marginally reduced (HR, 0.931; 95% CI, 0.821-1.057). The trend across age groups was significant (*P* < 0.001). It remained consistent even when multiple variables were adjusted for in successive models.

**Table 3:** Hazard ratios and 95% confidence intervals for association of age at menopause and risk of suicide.

Age at menopause (years)	Subjects (*n*)	Events (*n*)	Follow-up duration (person-year)	Incidence rate^a^	Hazard ratio (95% Confidence interval)					
					Model 1^b^	Model 2^c^	Model 3^d^	Model 4^e^	Model 5^f^	Model 6^g^
< 40	23452	83	275033.71	0.30	1.72 (1.38-2.15)	1.49 (1.20-1.86)	1.46 (1.17-1.82)	1.44 (1.16-1.80)	1.43 (1.14-1.78)	1.42 (1.14-1.77)
40-44	77099	242	906165.88	0.27	1.52 (1.33-1.75)	1.36 (1.19-1.56)	1.34 (1.17-1.53)	1.32 (1.15-1.52)	1.31 (1.15-1.50)	1.30 (1.14-1.49)
45-49	360032	866	4304249.6	0.20	1.15 (1.06-1.25)	1.16 (1.07-1.26)	1.15 (1.06-1.25)	1.14 (1.05-1.24)	1.13 (1.04-1.23)	1.13(1.04-1.23)
50-54	718110	1506	8603871.93	0.18	1 (Ref.)	1 (Ref.)	1 (Ref.)	1 (Ref.)	1 (Ref.)	1 (Ref.)
≥ 55	137102	289	1647558.32	0.18	1.00 (0.88-1.14)	0.92 (0.81-1.04)	0.92 (0.82-1.05)	0.94 (0.82-1.06)	0.93 (0.82-1.06)	0.94(0.82-1.06)
*P* value					<0.001	<0.001	<0.001	<0.001	<0.001	<0.001
*P* for trend					<0.001	<0.001	<0.001	<0.001	<0.001	<0.001

a Incidence per 1000 person-years.

b Model 1 was not adjusted.

c Model 2 was adjusted for age.

d Model 3 was adjusted for age- income- smoking status- alcohol consumption and physical activity.

e Model 4 was adjusted for age- income- smoking status- alcohol consumption- physical activity- obesity- type 2 diabetes- hypertension- dyslipidemia- chronic kidney disease and depression.

f Model 5 was adjusted for age- income- smoking status- alcohol consumption- physical activity- obesity- type 2 diabetes- hypertension- dyslipidemia- chronic kidney disease- depression- age at menarche- parity- breastfeeding- use of oral contraceptives- hormone therapy

g Model 6 was adjusted for age- income- smoking status- alcohol consumption- physical activity- obesity- type 2 diabetes- hypertension- dyslipidemia- chronic kidney disease- depression- age at menarche- parity- breastfeeding- use of oral contraceptives- hormone therapy- schizophrenia and anxiety.

### 3.4 Subgroup analysis

In the subgroup analysis shown in **Table 4**, HRs for suicide risk associated with age at menopause were calculated after adjusting for demographic, lifestyle, and health-related variables. The analysis revealed that across subgroups (categorized by age, income level, smoking status, alcohol consumption, physical activity, obesity, type 2 diabetes, hypertension, dyslipidemia, chronic kidney disease, depression, age at menarche, parity, breastfeeding, and use of oral contraceptives or hormone therapy), the relationship between earlier age at menopause and increased suicide risk remained consistent. Results showed no significant differences in this association among different subgroups based on these factors (*P* for interaction > 0.05 for all subgroups).

**Table 4:** Subgroup analysis of suicide risk by age at menopause.

Subgroups		Age at Menopause (years)					
		<40	40-44	45-49	50-54	55-	*P* for interaction
Age (years)	30-64	1.80 (1.25-2.57)	1.42 (1.13-1.78)	1.11 (0.98-1.26)	1 (Ref.)	0.95 (0.79-1.14)	0.464
	65-	1.25 (0.94-1.65)	1.24 (1.04-1.47)	1.14 (1.02-1.28)	1 (Ref.)	0.92 (0.78-1.10)	
Income^a^	Other	1.30 (1.00-1.68)	1.37 (1.18-1.59)	1.13 (1.03-1.24)	1 (Ref.)	0.94 (0.81-1.08)	0.340
	Low (Q1)	1.94 (1.27-2.96)	1.09 (0.79-1.51)	1.15 (0.96-1.38)	1 (Ref.)	0.92 (0.70-1.21)	
Smoking status^b^	No	1.46 (1.16-1.84)	1.37 (1.19-1.57)	1.13 (1.04-1.23)	1 (Ref.)	0.94 (0.83-1.07)	0.173
	Yes	1.08 (0.47-2.47)	0.61 (0.31-1.22)	1.16 (0.83-1.61)	1 (Ref.)	0.69 (0.36-1.33)	
Alcohol consumption^c^	No	1.40 (1.11-1.78)	1.28 (1.11-1.48)	1.11 (1.02-1.22)	1 (Ref.)	0.96 (0.84-1.09)	0.298
	Yes	1.63 (0.86-3.08)	1.56 (1.06-2.28)	1.30 (1.02-1.64)	1 (Ref.)	0.70 (0.45-1.08)	
Regular exerciser^d^	No	1.50 (1.19-1.89)	1.26 (1.09-1.47)	1.12 (1.02-1.23)	1 (Ref.)	0.94 (0.81-1.08)	0.447
	Yes	0.94 (0.44-2.00)	1.62 (1.16-2.26)	1.21 (0.98-1.49)	1 (Ref.)	0.92 (0.68-1.24)	
Obesity^e^	No	1.50 (1.15-1.96)	1.35 (1.15-1.59)	1.13 (1.02-1.25)	1 (Ref.)	0.89 (0.75-1.05)	0.747
	Yes	1.28 (0.86-1.91)	1.23 (0.97-1.57)	1.15 (0.99-1.33)	1 (Ref.)	1.00 (0.82-1.22)	
Type 2 diabetes	No	1.45 (1.14-1.85)	1.39 (1.20-1.60)	1.13 (1.03-1.24)	1 (Ref.)	0.92 (0.80-1.06)	0.428
	Yes	1.33 (0.79-2.25)	0.98 (0.68-1.41)	1.17 (0.95-1.44)	1 (Ref.)	0.99 (0.74-1.31)	
Hypertension	No	1.57 (1.15-2.14)	1.32 (1.08-1.60)	1.12 (0.99-1.25)	1 (Ref.)	0.89 (0.74-1.07)	0.857
	Yes	1.31 (0.96-1.80)	1.31 (1.08-1.58)	1.15 (1.02-1.30)	1 (Ref.)	0.97 (0.82-1.15)	
Dyslipidemia	No	1.38 (1.04-1.82)	1.32 (1.20-1.57)	1.15 (1.03-1.28)	1 (Ref.)	0.94 (0.80-1.11)	0.965
	Yes	1.52 (1.07-2.17)	1.29 (1.02-1.63)	1.10 (0.96-1.27)	1 (Ref.)	0.91 (0.74-1.12)	
Chronic kidney disease	No	1.49 (1.17-1.90)	1.34 (1.15-1.56)	1.14 (1.04-1.25)	1 (Ref.)	0.92 (0.80-1.06)	0.863
	Yes	1.17 (0.68-2.00)	1.20 (0.87-1.65)	1.11 (0.91-1.37)	1 (Ref.)	0.99 (0.73-1.33)	
Depression	No	1.60 (1.25-2.03)	1.29 (1.10-1.51)	1.14 (1.04-1.26)	1 (Ref.)	0.92 (0.80-1.07)	0.423
	Yes	0.91 (0.52-1.59)	1.39 (1.06-1.82)	1.11 (0.93-1.32)	1 (Ref.)	0.96 (0.74-1.25)	
Age at menarche (years)	<13	.	1.25 (0.28-5.62)	1.77 (0.77-4.09)	1 (Ref.)	1.58 (0.44-5.66)	0.792
	13-14	1.48 (0.72-3.00)	1.73 (1.19-2.52)	1.28 (1.00-1.64)	1 (Ref.)	0.95 (0.62-1.43)	
	15-16	1.87 (1.28-2.73)	1.24 (0.96-1.61)	1.09 (0.94-1.26)	1 (Ref.)	0.88 (0.70-1.10)	
	17-	1.24 (0.93-1.67)	1.26 (1.05-1.51)	1.12 (1.00-1.25)	1 (Ref.)	0.94 (0.80-1.11)	
Parity	Nulliparity	3.12 (1.21-8.02)	1.31 (0.55-3.14)	1.57 (0.95-2.60)	1 (Ref.)	1.43 (0.66-3.10)	0.384
	≥1	1.38 (1.10-1.74)	1.31 (1.14-1.51)	1.12 (1.03-1.22)	1 (Ref.)	0.92 (0.81-1.05)	
Breastfeeding	No	1.74 (0.81-3.74)	1.00 (0.56-1.79)	1.24 (0.90-1.67)	1 (Ref.)	1.40 (0.90-2.18)	0.263
	Yes	1.41 (1.12-1.77)	1.34 (1.16-1.54)	1.13 (1.03-1.23)	1 (Ref.)	0.90 (0.79-1.03)	
OC	No	1.36 (1.06-1.74)	1.29 (1.11-1.50)	1.17 (1.07-1.28)	1 (Ref.)	0.94 (0.82-1.08)	0.237
	Yes	1.86 (1.11-3.14)	1.42 (1.01-2.00)	0.95 (0.76-1.18)	1 (Ref.)	0.89 (0.66-1.20)	
HRT	No	1.52 (1.20-1.92)	1.36 (1.17-1.57)	1.16 (1.06-1.27)	1 (Ref.)	0.93 (0.81-1.06)	0.419
	Yes	0.92 (0.45-1.85)	1.08 (0.73-1.57)	1.01 (0.82-1.25)	1 (Ref.)	0.95 (0.71-1.28)	

Adjusted Hazard ratios (HRs) and 95% CI for suicide associated with age at menopause in subgroups (values were calculated after adjusting for age, income, smoking status, alcohol consumption, physical activity, obesity, type 2 diabetes, hypertension, dyslipidemia, chronic kidney disease, depression, age at menarche, parity, breastfeeding, use of oral contraceptives, and menopausal hormone therapy).

BMI, body mass index; BP, blood pressure; HRT, hormone replacement therapy; OC, oral contraceptives.

^a^Income: Individuals in the bottom 25% income bracket based on health insurance premiums in South Korea are identified as low income.

^b^Smoking status: Includes individuals who currently smoke, as opposed to non-smokers (never-smokers and ex-smokers).

^c^Alcohol consumption: Individuals reporting any frequency of alcohol consumption.

^d^Regular Exerciser: Individuals engaging in moderate-intensity exercise for at least 30 minutes on 5+ days per week, or vigorous activity for at least 20 minutes on 3+ days per week.

^e^Obesity: Body mass index (BMI) of 25 kg/m^2^ or higher.

## 4 Discussion

This study examined the association between age at menopause and risk of suicide. Our analysis yielded two primary findings (1): women who experienced menopause before the age of 40 exhibited a significantly increased suicide risk, and (2) this risk progressively decreased with increasing age at menopause, showing a potential dose-response relationship. These findings remained robust after adjusting for demographic, lifestyle, clinical, and reproductive factors, underscoring the impact of menopausal timing on women’s mental health. Subgroup analyses supported these associations across different demographic and health-related variables including depression status.

Our results align with previous studies indicating increased mortality and psychiatric disorders in women experiencing POI or early menopause (15). However, they diverge from a Japanese study that found no link between age at menopause and suicide risk, indicating potential demographic or regional differences (34). These mixed findings highlight the importance of considering population-specific factors in understanding menopausal mental health outcomes.

This study provides a novel perspective by examining completed suicide risk rather than suicidal ideation alone, which has been the primary focus of previous research (19). For instance, a cross-sectional study has typically linked early menopause and POI to an increased risk of suicidal ideation but lacked longitudinal follow-up to assess completed suicide as an outcome (19). Our longitudinal approach contributes essential evidence on the cumulative impact of early menopause on mental health, complementing findings from studies focused on ideation.

This study further contributes to understanding menopause-related mental health beyond depression, as early menopause has traditionally been associated with elevated depression risk (16, 35, 36). While depression is a widely recognized risk factor for suicide (22), our findings indicate a direct link between early menopause and elevated suicide risk independent of depression, consistent with a previous study showing genetic correlations with suicide attempts (37). Additionally, a study on Korean women with POI reports a heightened risk of suicidal ideation regardless of major depressive disorder diagnosis (19). This indicates that the mental health implications of early menopause extend beyond depression and may involve other mechanisms.

The increased suicide risk associated with early menopause may be partially attributable to hormonal changes. The transition into menopause brings physiological changes, particularly alterations in neurosteroids such as estrogen and progesterone, which are vital to neuroendocrine regulation, mood stability (38), and control of various behavioral processes (39). Estrogen impacts mood and cognitive functions through its interactions with neurotransmitters neuroprotective mechanisms (40, 41). Estrogen has been associated with a decreased risk of neurodegenerative diseases in postmenopausal women. Its neuroprotective effect is attributed to its activation of estrogen receptors, modulation of gene transcription, and antioxidant effects (42–44). These neurobiological processes are critical for understanding its comprehensive influence on mitigating mood disorders and cognitive decline post-menopause (45, 46). Progesterone plays a crucial role in the central nervous system during menopause. It impacts cognitive processes, mood regulation, inflammation, neurogenesis, and recovery (47), linking hormonal changes to reproductive mood disorders (48). Additionally, progesterone contributes to neural homeostasis, neuroprotection (49), and the development of oligodendrocytes, which are essential for myelination and cognitive function (50). Abrupt hormonal changes during early menopause could disrupt established neuroendocrine balance and neural functions, potentially aggravating mental health conditions during this critical transition period (51). In our study, we included various clinical variables and reproductive factors such as parity, breastfeeding duration, as well as the use of HRT or OC, as these can affect cumulative estrogen and progesterone exposure (23, 24, 26). Our findings remained robust after accounting for these variables, suggesting that menopausal timing itself plays a distinct role in suicide risk beyond the effects of reproductive history and mental health status.

Furthermore, menopause entails profound psychosocial effects. Women may experience a sense of loss related to declining fertility, changes in physical health, and societal attitudes toward aging (52). The stigma surrounding menopause and the perceived loss of youth and fertility can lead to decreased self-worth and identity crises, which are risk factors for mental health issues (18, 53). For those with POI, these experiences can be more intense due to a premature onset, leading to a potentially greater psychosocial impact and a risk of developing mental health conditions predisposing to suicidal ideation and behavior (15). These psychosocial factors likely interact with the biological changes occurring during early menopause, compounding mental health vulnerabilities. This underscores the complex interaction between hormonal changes, reproductive history, and mental health in influencing suicide risk, reinforcing the need for a comprehensive approach to managing mental health in women experiencing early menopause.

Our study utilized the NHIS database of over 52 million individuals with national coverage and long follow-up (27). This database offers robust statistical power and a wide array of data, enhancing the validity and reliability of our findings (29). The incorporation of a wide range of reproductive and health-related factors in our analysis provides a nuanced understanding of the relationship between menopausal timing and suicide risk. However, our study has limitations, including its retrospective nature that precludes the establishment of the causality between menopausal timing and suicide risk. It has potential for recall bias and misclassification due to reliance on self-reported data. Additionally, the findings may be subject to selection bias and are primarily generalizable to Korean women, limiting their applicability to other populations. Furthermore, the identification based on ICD-10 codes in medical records might not fully capture nuances of mental health conditions as this approach does not include systematic assessments of mental health. Cultural factors specific to Korea, such as stigma or reluctance to seek psychiatric help (54–56), might have led to underreporting or underdiagnosis of mental health conditions. Certain confounders, such as gravidity and psychological factors influencing early menopause (e.g., cognitive status, perceived stress), were not available, which may contribute to residual confounding. Also our study did not account for genetic predispositions or autoimmune conditions, which are known to influence both menopausal timing (12) and mental health outcomes. Future studies could benefit from incorporating a broader range of psychological, genetic, autoimmune, and reproductive factors to provide a more comprehensive view of the relationship between menopausal timing and suicide risk. To expand our understanding of menopause’s impact on mental health, longitudinal studies with diverse populations and data including precise menopause onset dates and extensive clinical histories are essential to validate our findings and enhance their relevance across different demographic groups. Exploring psychosocial aspects such as societal perceptions and individual coping strategies will provide further insight into postmenopausal mental health challenges.

Our research highlights the necessity of a proactive, integrated clinical approach to address the suicide risk associated with early menopause and POI. Healthcare providers should implement targeted risk assessments and regular screenings. Timely interventions are crucial for identifying and managing mental health issues effectively. While HRT is a recommended treatment (57), its application requires caution due to potential risks such as increased depression, especially after initiation (35, 58). A comprehensive approach combining cautious use of HRT, psychoeducation, and psychological support is vital for preventing suicide and enhancing the overall well-being of women undergoing early menopause or POI (18, 59).

In conclusion, findings of this study suggest that postmenopausal women who experience menopause at an earlier age, particularly those with POI, may face an increased risk of suicide. Such insight into the relationship between menopausal age and suicide risk emphasizes the need for incorporating reproductive health factors into mental health assessments and suicide prevention strategies for postmenopausal women.

## Data Availability

Publicly available datasets were analyzed in this study. This data can be found here: https://nhiss.nhis.or.kr/.
